# Recent Advances in the Development of Pro-PROTAC for Selective Protein Degradation

**DOI:** 10.3390/pharmaceutics17091160

**Published:** 2025-09-04

**Authors:** Fady Hakem, Ahmad Abdelwaly, Reem Alshaman, Abdullah Alattar, Fawaz E. Alanazi, Sawsan A. Zaitone, Mohamed A. Helal

**Affiliations:** 1 Biomedical Sciences Program, University of Science and Technology, Zewail City of Science and Technology, Giza 12587, Egypt; p-fady.hakem@zewailcity.edu.eg; 2 Institute for Computational Molecular Science, Department of Chemistry, Temple University, Philadelphia, PA 19122, USA; abdelwaly@temple.edu; 3 Department of Pharmacology and Toxicology, Faculty of Pharmacy, University of Tabuk, Tabuk 47512, Saudi Arabia; ralshaman@ut.edu.sa (R.A.); aalattar@ut.edu.sa (A.A.); falanazi@ut.edu.sa (F.E.A.); szaitone@ut.edu.sa (S.A.Z.); 4 Medicinal Chemistry Department, Faculty of Pharmacy, Suez Canal University, Ismailia 41522, Egypt

**Keywords:** PROTAC, ubiquitin, thalidomide, degradation, opto-PROTACs, proteasome

## Abstract

PROTACs are trimeric small molecules consisting of a specific modulator of the target protein connected to a ligase-recruiting ligand via a suitably flexible linker. Ligase-recruiting ligands deliver ubiquitin ligases like E3 ligase to the Protein of Interest (POI). The vicinity of the POI-PROTAC-E3 ternary complex enables the E3 ligase to ubiquitinate the surface lysine residues of the POI. The Ubiquitin–Proteasome System (UPS) then degrades the POI. However, despite the considerable advances in the design of PROTACs targeting several types of enzymes and receptors, this strategy is still facing the challenges of precision target delivery and duration of action. In this review, we highlight the recent approaches for the development of PROTAC prodrugs or pro-PROTAC to control the delivery of PROTACs and achieve the required on-target exposure. This strategy may facilitate the application of the PROTAC technology and expand its clinical benefits.

## 1. Introduction

Proteolysis Targeting Chimera (PROTAC) technology is a revolutionary approach designed to induce protein degradation. It offers a unique solution to deal with the limitations of traditional small-molecule drugs. The PROTAC concept was developed by the Crews and Deshaies groups in 2001. Since then, it has been efficiently employed to target various functional proteins with different subcellular localizations, especially in the degradation of several kinases implicated in cancer. PROTACs were found advantageous in overcoming resistance induced by the protein target overexpression or mutation compared to small molecule inhibitors [1,2,3,4].

**Figure 1:**
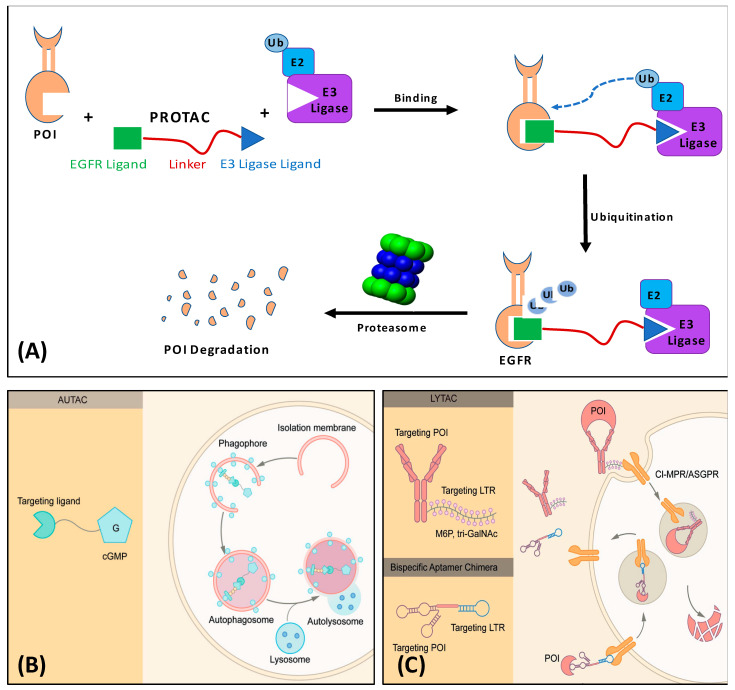
(**A**) Schematic illustration of the PROTAC approach for protein degradation. PROTAC consists of a POI warhead (EGFR, for example) linked to an E3 ligase ligand; (**B**) AUTAC degraders consist of a POI-tag, a linker, and a cGMP-based ligand; (**C**) LYTAC is composed of a ligand or an antibody connected to a molecule that binds to lysosome-targeting receptors (LTRs). The LTR molecule here is tri-GalNAc. (**B**,**C**) are adopted from *Signal Transduction and Targeted Therapy* (2022) 7:113 [21], under Creative Common license.

PROTACs consist of three main components: a ligand that binds to the target protein or the Protein of Interest (POI), an E3 ligase-binding ligand, and a linker connecting the two moieties (Figure 1). Notably, the linker plays a crucial role in the degradation efficiency of PROTACS, and its optimization is a significant challenge in developing these molecules [5,6,7]. PROTACs are designed to recruit an E3 ubiquitin ligase to mark a specific target protein for degradation by the cellular proteasome system. This strategy has the potential to modulate disease-associated proteins that have been difficult to target with traditional small-molecule inhibitors. The development of PROTAC opened the door for inspiration for developing other protein-degrading molecules by hijacking various biological systems. Autophagy-Targeted Chimeric complexes (AUTACs) and Autophagosome-Tethering Chimeras (ATTECs) are also trimeric molecules that use intracellular autophagy machinery to facilitate the degradation of target proteins; however, they rely on a system different from the ubiquitin-proteasome system (UPS). Notably, these strategies are restricted to cytosolic proteins due to the cytosolic localization of the target autophagy machinery, which also requires that degraders are permeable to the cell [8,9]. Also interesting is the Lysosome Targeting Chimera (LYTAC), which is a good complement to PROTACs as it induces the degradation of extracellular and membrane proteins via the endosome–lysosome system. This trimeric molecule can connect the POI to a lysosome-targeting receptor (LTR) found on the cellular surface, leading to protein internalization via clathrin-mediated endocytosis. This strategy was introduced in 2019 with no candidates in clinical trials to date. Nevertheless, LYTAC holds great potential as a tool to target membrane proteins such as G-Protein Coupled Receptors (GPCRs) and receptor tyrosine kinases [10,11,12].

As of the date of this review, there are no FDA-approved PROTAC drugs on the market. However, these hybrid molecules have demonstrated unique advantages in addressing several drug targets, and some representative PROTACs are being studied in clinical trials for the treatment of cancers and other diseases, such as neurodegenerative and immune system disorders [13,14,15]. Currently, there are over 30 PROTAC candidates actively investigated in different stages of clinical trials. The targeted proteins include androgen receptor (AR), estrogen receptor (ER), Signal transducer and activator of transcription 3 (STAT3), Bruton’s tyrosine kinase (BTK), and interleukin-1 receptor-associated kinase 4 (IRAK4) [16]. Collectively, there are 19 PROTACs in phase I, 12 in phase II, and 3 in phase III. The clinical trials of the most advanced PROTAC drugs, ARV-110 and ARV-471 from Arvinas, have shown encouraging results for prostate and breast cancer, respectively [14,17,18,19,20]. Notably, Artificial intelligence (AI) is also revolutionizing the design of bioactive PROTACs through predictive modeling and molecular simulations. Recently, several predicted AI PROTAC models were reported and applied for the design of potent protein degraders. For example, AIMLinker, a deep encoder–decoder neural network, generates novel linker moieties by extracting structural fragment information and filtering out non-druggable candidates [21]. In addition, ShapeLinker implements fragment-linking using reinforcement learning on a SMILES generator to design PROTAC linkers [22]. Concerning the predictive models, DeepPROTAC feeds ligands and binding pockets into GCNs for feature extraction. Then, linkers are represented using SMILES notation to generate features for rational PROTAC design [23].

PROTACs significantly reduce multiple genetic or phenotypic resistances, which are typically developed by protein targets against small molecules through mutation and overexpression. An example is the resistance developed by the androgen receptor against Enzalutamide. In addition, PROTACs possess additional advantages related to their effective therapeutic concentration. There is a need to sustain a specific concentration of the traditional small molecule inhibitors (SMIs) to achieve a sufficient level of occupancy at the active site of a protein of interest (POI), eventually interfering with the biological process. In contrast, the targeted protein ubiquitination and subsequent proteasomal degradation are catalytic in nature, which permits a minor concentration of PROTACs to elicit enhanced biological activities [24,25,26,27,28]. Due to the above-mentioned advantages, compared to traditional drugs, PROTACs managed to grasp the attention and to become one of the hot areas of drug development research.

Despite the prominent advantages of PROTACs, they have a number of disadvantages. First, PROTACs are less selective or non-specific to the malformed cells or the disease-causing proteins, leading to some side effects and toxicity. Thus, careful consideration is needed while designing novel PROTACs to precisely target the proteins implicated in the disease. Second, low expression and/or mutation of the recruited E3 ligase may lead to resistance of the target protein to degradation, negatively influencing the PROTAC efficacy. Latent PROTACs or pro-PROTACs are protected with various labile groups that can be selectively removed under certain physiological or experimentally induced conditions, releasing the biologically active form. PROTAC latentiation could serve the following purposes: (1) selective targeting of oncogenic proteins; (2) prolonging the biological action; (3) selectively investigating poorly studied protein signaling pathways in vitro. The development of PROTACs has been extensively reviewed in recent literature. However, our review focuses on the emerging strategies to develop “prodrugs” of PROTAC (pro-PROTAC). These strategies address the inherent limitations by offering a controlled release of PROTAC molecules, thereby enhancing on-target exposure and potentially expanding the clinical application of this groundbreaking technology. Notably, only a few reviews covered the concept of PROTAC prodrugs, especially for tuning the on-target effect and avoiding toxicity [6,29,30,31,32,33,34]. In this review, we will cover the recent advances in the field of the development of PROTACs prodrugs, including those designed for controlling the target selectivity, for prolonging the biological action, or for any other purposes [35].

## 2. Photocaged PROTACs (opto-PROTACs)

**Figure 2:**
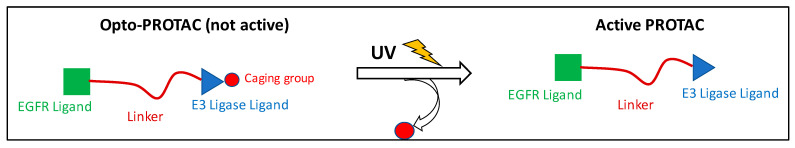
Mechanism of activation of the opto-PROTACs.

One of the most interesting approaches for latentiation is the design of photocleavable PROTACs, which are protected or “caged” with a photolabile group such as 4,5-dimethoxy-2-nitrobenzyl moiety (DMNB). In this approach, a light with a specific wavelength is used to activate and release the functional PROTACs from the caged ones (Figure 2) [36]. This technique allows the spatiotemporal control of proteolysis. These pharmacological tools should be cell-permeable, primarily inert as degraders, and stable intracellularly before light activation.

**Figure 3:**
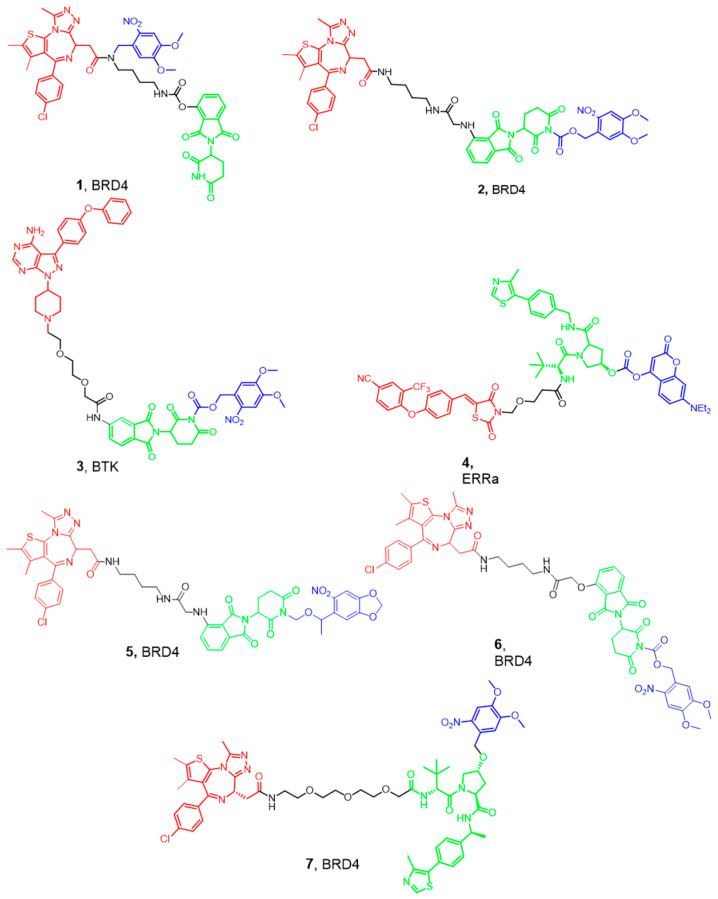
Examples of the recently developed Photo-caged PROTACs. Compounds are composed of a POI-binding moiety (red), a linker (black), an E3 ligase-binding moiety (green), and a caging moiety removed by UV (blue). The POI of each PROTAC is mentioned with the molecule number.

PROTAC caging is mostly achieved by installing a photolabile group on the −NH of the glutarimide group of the Cereblon (CRBN) ligand or the hydroxyl group of the Von Hippel–Lindau (VHL) ligand. This moiety should prevent the critical H-bond interactions with the corresponding E3 ligase, thus inhibiting the formation of the ternary complex needed for ubiquitination of the protein of interest. Another strategy is to install the caging group on the ligand targeting the POI, hindering the biological target engagement. One of the first examples of photocaged PROTACs is the work by Xue et al., who used the DMNB group to develop BRD4-targeting photocaged degraders (compounds **1** and **2**) (Figure 3) [37]. Irradiation with UV light at 365 nm resulted in the liberation of the active PROTAC and a dose-dependent BRD4 degradation in a zebrafish embryo model. They have also developed a BTK opto-PROTAC by installing the same DMNB group onto the glutarimide NH, obtaining a light-inducible protein degradation in Ramos cells (compound **3**). Later, scientists from the University of Pittsburgh installed a diethylamino coumarin (DEACM) caging group onto the hydroxyproline moiety of VHL using a carbonate linkage to develop the estrogen receptor α (ERRα) degrader 4. Similarly, they attached the 6-nitropiperonyloxymethyl (NPOM) group to the glutarimide nitrogen of thalidomide to generate the latent BRD4 PROTAC 5. The authors demonstrated that subsequent light activation resulted in the removal of the DEACM and NPOM moieties, restoring the degrader activity, and leading to an efficient antiproliferative activity in the 22Rv1 cells [36]. In the same year, researchers from Harvard Medical School installed a DMNB group onto the glutarimide nitrogen of the pomalidomide moiety of a PROTAC based on dBET1 (compound **6**). This molecule was able to produce a dose-dependent degradation of BRD3 and BRD4 upon irradiation with UV light at 365 nm [38]. Similarly, Kounde and co-workers used the popular DMNB group to mask the critical hydroxy group of the VHL ligand, generating the caged PROTAC 7 targeting BRD4. Irradiation at 365 nm resulted in the degradation of green fluorescent protein (GFP)-tagged BRD4 in HEK293 cells [39]. This approach would allow masking the cytotoxic effect of PROTACs derived from BRD4 inhibitors intracellularly. Exposure to UV light with the appropriate wavelength could release the active form of the cytotoxic PROTAC at the tumor site. This strategy possesses great potential in targeting skin diseases but would be of limited value in deeper sites.

## 3. In-Cell Click-Formed Proteolysis-Targeting Chimeras (CLIPTAC)

The properties of current PROTACs may limit their potential use for protein degradation in vivo. For instance, the incorporation of both a target protein-ligand and an E3 ligase recruiting element leads to hetero-bifunctional molecules characterized by having high molecular weight and polar surface area, typically in the range 800–1000 Da and ~200 Å^2^, respectively [37,40]. Moreover, having high molecular weights can limit cellular permeation and solubility and compromise bioavailability and pharmacokinetics, especially distribution to the CNS [41,42]. Furthermore, the length of the linker is important for target E3 ligase pairing. A short linker may sterically prevent the target protein and E3 ligase from simultaneously binding to the PROTAC, while an overly long linker may fail to bring ligase and target protein into a sufficient proximity to elicit ubiquitination [41]. Also, a pressing concern in drug development is achieving the required tissue selectivity, especially for PROTACs, which lack intrinsic tissue specificity, leading to potential off-target adverse effects [43]. 

**Figure 4:**
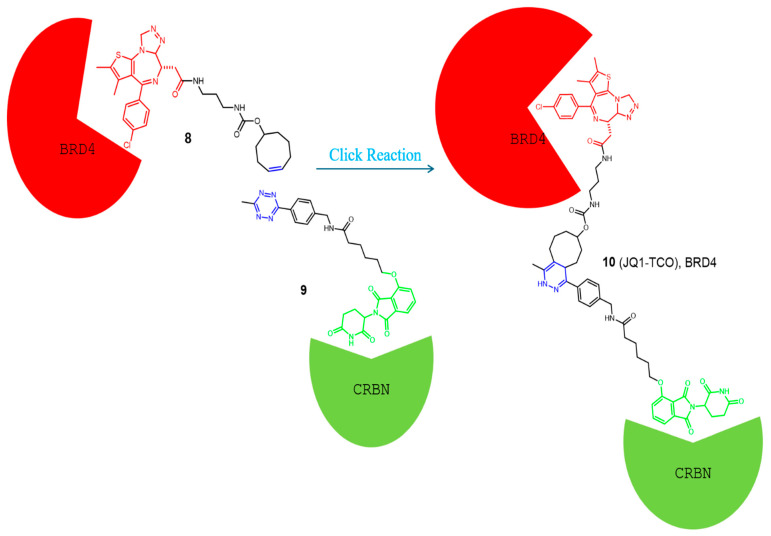
Example of a CLIPTAC developed by Lebraud et al. [41], and the click reaction occurs in vivo to degrade the BRD4 in cancer cells. Compounds are composed of a POI-binding moiety (red), a linker (black), and an E3 ligase-binding moiety (green). The substructure which undergoes the click reaction is colored blue in each compound.

To overcome these problems, in 2016, Lebraud and his colleagues developed a new strategy for the development of functional PROTACs [41]. Instead of administering the PROTAC as a trimeric molecule, they provided two low molecular weight separate fragments that can undergo a bio-orthogonal click reaction in vivo to yield the active PROTAC. From the reported reactions, the inverse electron demand Diels-Alder (IEDDA) cycloaddition between tetrazine and *trans*-cyclo-octene (TCO) grasped the researchers’ attention due to its rapidness, high yield, and occurrence without the presence of a catalyst [43,44]. Moreover, the reaction showed high specificity due to the absence of any cross-reactions, thus preventing the formation of non-selective molecules. The first fragment used was a tetrazine ring tagged with the thalidomide group needed for E3 ligase recruitment. A *trans*-cyclo-octene linked to a ligand for binding with the POI represents the second fragment. Based on these results and the high specificity of the reaction, they developed a CLIPTAC molecule (JQ1-TCO, 10) active against BRD4, as shown in Figure 4 [42]. Western blot studies showed a concentration-dependent degradation of BRD4 by JQ1–TCO. At 3 mM (t_1/2_ = 33 s) and 10 mM (t_1/2_ = 10 s) of JQ1-TCO together with Tz–thalidomide, complete degradation of BRD4 was noted, while at 0.3 mM (t_1/2_ = 5.5 min) and 1 mM (t_1/2_ = 100 s) of JQ1-TCO, partial degradation occurred.

**Figure 5:**
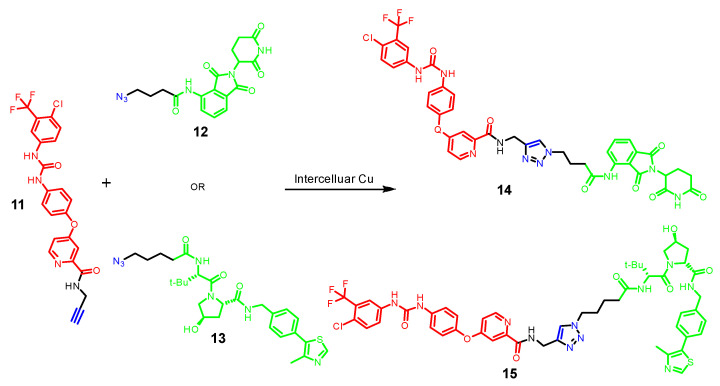
CLIPTACs depending on CuAAC assembly. Compounds are composed of a POI-binding moiety (red), a linker (black), an E3 ligase-binding moiety (green), and the two clickable groups (blue). The copper-catalyzed azide-alkyne cycloaddition (CuAAC) reaction is known for its high reaction rate, typically second-order with rate constants ranging from 10 to 10^4^ M^−1^s^−1^.

Copper (Cu) is a vital element for humans with an essential role in cell proliferation and angiogenesis. Studies showed that Cu concentration was 46% higher in certain cancer tissues than in the normal ones. Thus, tumor-targeted therapies based on this differential tissue distribution have been developed [45]. In this regard, Si et al. developed tumor-selective PROTACs that are self-assembled intracellularly based on a Cu-mediated Azido-Alkyne Cycloaddition (CuAAC) reaction in the presence of Cu^+1^ (Figure 5). Specifically, alkyne-modified Sorafenib (11), a kinase inhibitor with multiple targets such as Vascular Endothelial Growth Factor Receptor (VEGFR-2) and Receptor Tyrosine Kinase (EphB4), and the azido-modified VHL and CRBN ligand (compounds **12** and **13**, respectively) were developed and successfully used for the synthesis of tumor-selective PROTACs assembled in situ via the CuAAC reaction (compounds **14** and **15**, respectively) [46,47]. HPLC and Ultrafast high-resolution mass spectrometry indicated the formation of self-assembled PROTAC intracellularly in both normal (A549) and tumor (HEK293) cells. Moreover, the cytotoxicity of the lead compounds and the combination of these agents towards A549 and HEK293 cells was measured by the MTT assay. The combination of precursor molecules had a significant effect on the cell viability of A549 cells; however, with no obvious cytotoxicity to HEK293 cells at 1 mM.

## 4. Folate PROTAC

As mentioned above, a downside of certain PROTACs is their lack of selectivity towards the biological target of interest, leading to the degradation of both healthy and defective proteins inside the cells. This is sometimes inherent in the design due to the poor selectivity of the small molecule ligand used to target the POI [48]. However, this selectivity could be achieved by exploiting the high expression of certain receptors on tumor cells and their corresponding endogenous ligands without modifying the ligand targeting the POI.

**Figure 6:**
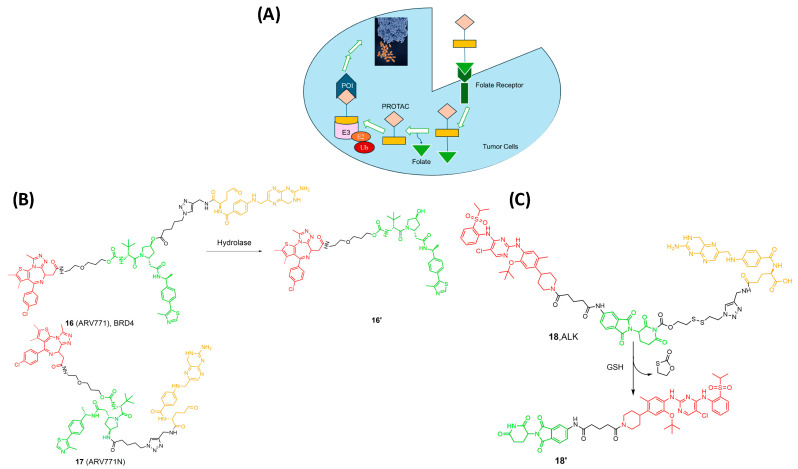
(**A**) Mechanism of Activation of Folate-PROTACs inside cancer cells for degradation of the POI; (**B**) Folate PROTAC based on the VHL ligand; (**C**) Folate-PROTAC based on Pomalidomide. Compounds are composed of a POI-binding moiety (red), a linker (black), an E3 ligase-binding moiety (green), and a cleavable folate ligand (orange).

Researchers have identified the folate receptor (FOLR1) as a biomarker for tumor cells in ovarian, breast, and lung cancers [32,49]. This membrane protein is responsible for binding with folic acid and its reduced derivatives, which are accountable for the intercellular delivery of tetrahydrofolate. Other receptors, such as FOLR2 and FOLR3, can act as transporters of folate across cell membranes, despite having a lower affinity to folate than FOLR1 [48,50]. Therefore, FOLR1-targeting has long been used as an effective strategy for cancer imaging and selective targeting of tumors, and several FOLR1-targeting drugs are in phase II/III clinical trials [51]. Installing the folate onto the E3 ligase ligand via a linker allows the selective uptake of the PROTAC by the cancer cells, followed by cleavage of the linker by endogenous hydrolases, releasing the active drug (Figure 6A) [52,53,54]. With this knowledge, Jin and Wei at Harvard University developed a series of folate PROTAC molecules based on a VHL ligand to target BRD [54,55]. From these compounds, ARV-771 (16) showed the greatest potency against the POI. Interestingly, folate-ARV-771 degraded BRD4 as efficiently as ARV-771 in HeLa cells, while the non-cleavable negative control (17, folate-ARV-771N) produced no degradation. Continuing their work, they developed another series by installing the folate onto a CBRN ligand through a disulfide bond, which is glutathione sensitive, to target ALK kinase (18, Figure 6) [53,54,55]. This strategy provided a generalizable PROTAC platform for the selective degradation of proteins in cancer cells. Unfortunately, there is no in vivo data for folate-PROTACs to date, and their pharmacokinetic and pharmacodynamic properties are still unknown and not fully studied. Moreover, the use of these PROTACs is limited to the cells that are characterized by overexpression of the FOLR1 receptor [53].

## 5. Reactive Oxygen Species (ROS) PROTAC

Hydrogen peroxide, superoxide radicals, and hydroxyl radicals have an impact on oxidative stress and cellular signaling in various biological systems [32]. Their roles extend to influencing intracellular calcium levels, pH regulation, and even contributing to cardiac mechanical function during reperfusion ischemia. Furthermore, these molecules and radicals could be used to characterize tumor cells from normal cells, as they are highly expressed in cancer cells [32]. Therefore, PROTACs prodrugs activated by ROS may selectively target tumors with less toxicity to healthy tissues. The aforementioned opto-PROTAC requires radiation to be activated and remove the caging group that can affect the patient’s DNA and cause side effects. However, ROS is safer and more selective as it takes advantage of the microenvironment of tumor cells to undergo the activation process [56].

**Figure 7:**
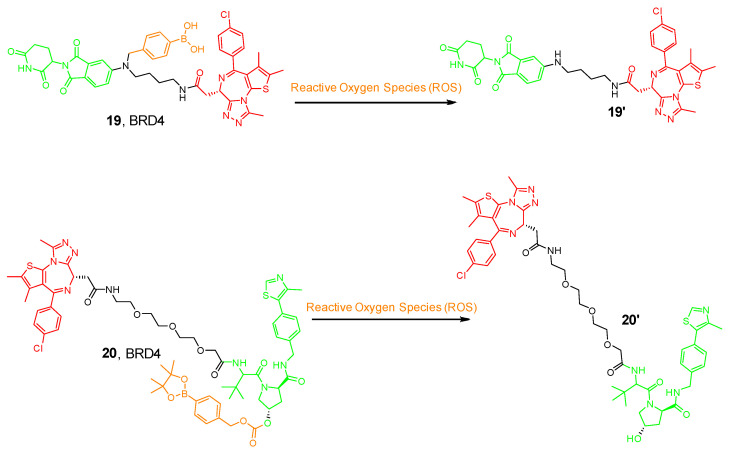
Example of the ROS-activated PROTAC compounds. Compounds are composed of a POI-binding moiety (red), a linker (black), an E3 ligase-binding moiety (green), and a cleavable ROS moiety (orange).

Scientists at ShanghaiTech University introduced a series of ROS-activated PROTACs that recruit CRBN E3 ligase for the selective degradation of BRD3. The prodrug contained an aryl boronic acid acting as a cage installed on the Lenalidomide ligand (19 in Figure 7) [56]. The aryl boronic acid cage transformed into a phenolic intermediate and underwent subsequent 1,6-benzyl exclusion to liberate the active PROTAC upon exposure to H_2_O_2_. Interestingly, this molecule selectively degrades BRD3 in tumor cells, whereas the parent PROTAC exhibits similar anti-proliferative activity in both cell types [56].

Furthermore, another research group from China designed a VHL-based pro-PROTAC where the hydroxy group of the VHL ligand was caged with an aryl boronic ester; thus, preventing the E3 ligase recruitment (20 in Figure 7) [57]. Cancer cells are characterized by high expression levels for NAD(P)H quinone dehydrogenase 1 (NQO1), a flavoenzyme responsible for two-electron reduction of quinones to hydroquinones. Researchers demonstrated that NQO1 can bioactivate antitumor compounds by forming unstable hydroquinones and producing ROS by redox cycling [57,58]. Therefore, NQO1-mediated reduction of β-lapachone (β-Lap) could allow an enhancement in the levels of cellular ROS [58]. Co-treatment with NQO1 and β-Lap with this pro-PROTAC molecule allowed the aryl boronic ester cage to be released, hence enabling the selective degradation of BRD4 in HeLa cells but not in 293T cells, expanding the chemical biology toolbox for cell-selective protein degradation.

## 6. Hypoxia-Activated PROTAC

**Figure 8:**
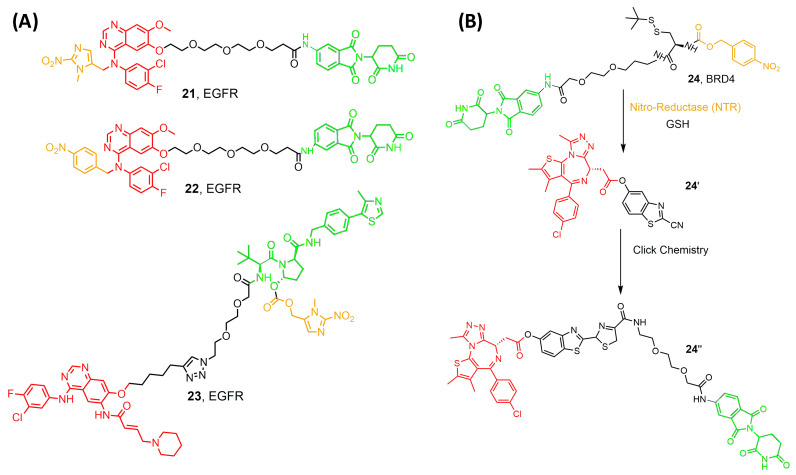
(**A**) compounds developed by Borad et al. [62] and Shi et al. [64] (**B**) compounds developed by Do et al. [65]. Compounds are composed of a POI-binding moiety (red), a linker (black), an E3 ligase-binding moiety (green), and a cleavable hypoxia moiety (orange).

Hypoxia is one of the solid tumor’s hallmarks and one distinguishing feature that occurs due to a mismatch in oxygen consumption and delivery [59]. Hypoxia encourages the overexpression of various carcinogenic proteins, such as HIF, VEGFR, and EGFR, that increase tumor resistance and survival rates. In the microenvironment of cancer cells, Nitroreductase (NT) is highly expressed, causing resistance against drugs (Figure 8A) [59,60]. Hypoxia could induce cell cycle arrest, and hypoxic tumor cells generally have a low proliferation rate, while radiotherapy and chemotherapy mainly act on proliferating cells. As a result, hypoxic tumor cells develop therapeutic resistance. Therefore, NTR-based prodrugs gain selectivity and differentiation between tumor and normal cells by this distinguishing feature [61]. Borad et al. developed the first NTR-prodrug Evofosfamide in 2015, showing safety and potency in clinical trials by installing a 2-nitroimidazole moiety [62]. Later, Cheng and his research group developed a series of hypoxia-activated PROTACs against epidermal growth factor (EGFR). They installed the hypoxia group on the 4-NH of the Gefitinib, leading to efficient degradation and selectivity towards EGFR^Del19^ (21 and 22) [63]. Interestingly, under normoxic conditions in HCC4006 cells, EFGR was not degraded, and UPLC-MS/MS analysis confirmed the stability of compounds, thus proving the selectivity of the compounds to cancer cells in hypoxic conditions [63]. Moreover, the same research group developed hypoxia-activated compounds with a different caging group. They introduced the (1-methyl-2-nitro-1H-imidazol-5-yl) methyl group into the EGFR ligand moiety of an EGFR^Del19^-targeting PROTAC. Unfortunately, the resulting compound targeted EGFR in both normal and cancer cells. This failed trial encouraged Shi and his colleagues to develop a series of hypoxia-activated PROTACs designed by incorporating the (1-methyl-2-nitro-1H-imidazol-5-yl) methyl group into the VHL E3 ligase recruiter (compound **23**) [64]. One of the developed compounds degraded EGFR in HCC-827 cells with an IC_50_ of 46.3 nM.

Recently, Do et al. introduced a novel enzyme-based clicking PROTACs (ENCTACs) strategy [65]. The release of the active PROTAC locally was achieved by orthogonally cross-linking the CRBN binder and BRD4 ligand upon the hypoxic activation with NTR and GSH (compound **24**). PROTAC degrades BRD4 selectively in several cancer cell lines, resulting in an accurate hypoxia-dependent activity in living cells, zebrafish, and mice with solid tumors [65]. This revolutionary approach may alleviate the adverse pharmacological effects observed with the conventional PROTACs, representing a great potential for developing a new treatment for cancer and other diseases.

## 7. Radiotherapy-Activated PROTAC (RT-PROTAC)

**Figure 9:**
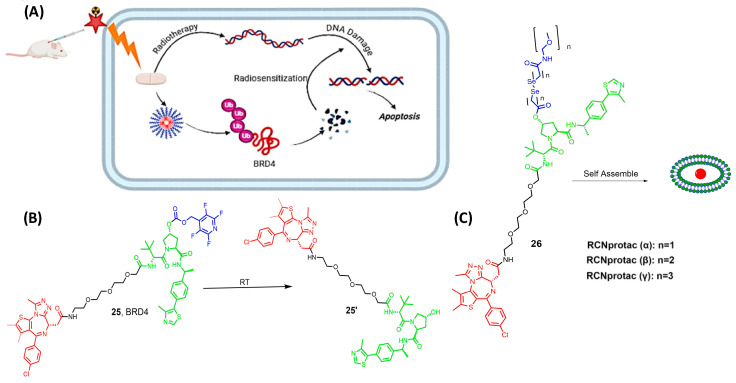
(**A**) mechanism of activation of RT-PROTAC, (**B**) compound developed by Yang and his research team, (**C**) compounds produced by Xu et al., depending on RCNprotac technology, where the length of the carbon chain differs. Compounds are composed of a POI-binding moiety (red), a linker (black), an E3 ligase-binding moiety (green), and the cleavable radioligand moiety (blue).

X-ray radiation therapy is a first-line clinical treatment for cancer [66]. Besides directly killing cancer cells via DNA damage, X-ray radiation is also widely used to excite sensitizers to generate reactive oxygen species and to trigger the drug release from either the prodrug or the nanoplatform, as previously mentioned [67,68,69,70,71]. However, the unexpected off-target protein degradation can restrict the application of PROTAC to various therapeutic conditions. In 2022, Yang and his research team successfully exploited X-ray radiation as a tool for PROTAC activation, where they used an X-ray cleavable caging group to mask the E3-ligase moiety (25 Figure 9) [72]. In the MCF-7 xenograft model, activated RT-PROTAC exhibits synergistic antitumor activity with radiation and degrades BRD4 and BRD2 equally as the free PROTAC degraders.

Xu et al. focused on developing an X-ray radiation-responsive PROTAC nanomicelle (RCNprotac) for tumor-specific proteolysis-enhanced radiotherapy. They aimed to integrate radiotherapy with protein degradation-mediated radiosensitization to prevent the repair of DNA and, thus, optimize anti-tumor therapy and avoid non-target toxicity [73]. MZ1 is one of the well-studied PROTACs with the ability to selectively degrade BRD4 over the other isoforms, BRD2 and BRD3 [74,75,76]. RCNprotac was synthesized by esterifying the hydroxyl group on the E3 ubiquitin ligand of MZ1 with a diselenide-bond-containing carbon chain and then conjugating PEG2000 to introduce hydrophilicity (26 Figure 9) [73]. The resulting amphiphilic RCNprotac precursor is self-assembled into nanomicelles. The conjugation of polyethylene glycol improves the solubility of the high molecular weight RCNportac, thus allowing ease in cell permeability and preventing the drawback of the high molecular weight [77]. The study results showed that the X-ray radiation-responsive PROTAC nanomicelles could effectively accumulate at the tumor site due to the enhanced permeability and retention effect. Upon exposure to X-ray radiation, the diselenide bonds in RCNprotac are broken, leading to the specific release of MZ1 for tumor BRD4 protein degradation. This degradation of BRD4 protein increased the tumor’s sensitivity to radiation, resulting in a synergistic enhancement of antitumor effects both in vitro and in vivo [73]. Moreover, the treatment with RCNprotac combined with X-ray radiation did not result in significant systemic side effects, indicating its potential as a safe and effective strategy for cancer therapy.

## 8. Semiconducting Polymer Nano-PROTAC (SPNpro)

**Figure 10:**
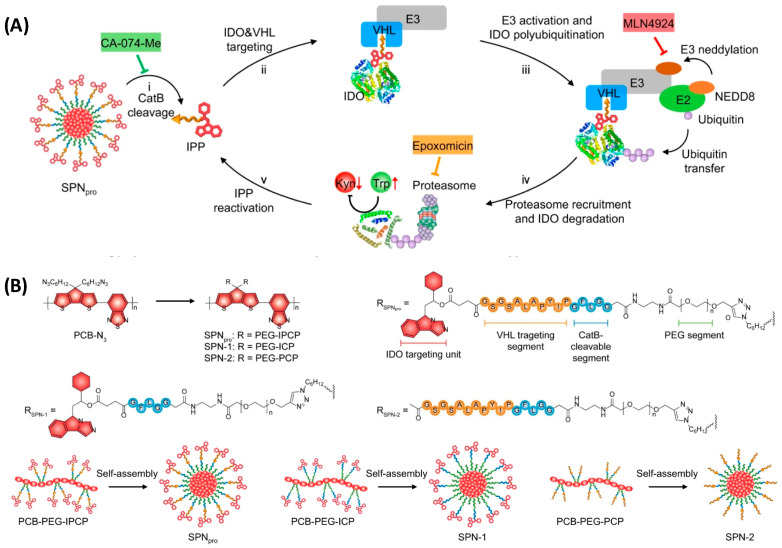
(**A**) Mechanism for CatB-specific activation of PROTAC-mediated IDO degradation; (**B**) The molecular structures and syntheses of SPNpro, SPN-1, and SPN-2. Figure adopted from *Nature Communications,* 2021, 12 (1): 2934 [78], under Creative Commons license.

Cancer immunotherapy has revolutionized the treatment of malignancies by activating innate and adaptive immune systems. However, fear of the development of immune-related adverse effects led to low patient response and restricted clinical trials [78,79]. The development of activatable immunostimulatory agents capable of responding to biomarkers in the tumor microenvironment is one of the ways to solve this issue [78]. However, the recently developed immunostimulatory agents take advantage of endogenous biomarkers associated with both tumor and normal cell microenvironments. Zheng et al. synthesized an amphiphilic semiconducting polymer self-assembled with a carbonylated NLG919, an indoleamine 2,3-dioxygenase (IDO) ligand [79,80]. The IDO was the protein of interest (POI) because it is a Tryptophan catabolizing enzyme causing the dysfunction of dendritic cells (DCs) and the inhibition of effector T cells through the exchange of Trp to kynurenine (Kyn) [78]. The researchers aimed to develop a novel approach that combines photodynamic therapy with biomarker-activated protein degradation to effectively suppress tumor progression in mouse models. They grasped the scientific community’s attention by representing a unique nano-PROTAC system offering a promising new strategy for cancer treatment. SPNpro serves as a nano-PROTAC for photo-immunometabolic therapy in cancer treatment. The specific design of SPNpro allows for the targeted proteolysis of immunosuppressive enzymes, such as indoleamine 2,3-dioxygenase (IDO), in response to the presence of the cancer biomarker Cathepsin B (CatB), which plays a crucial role in the activation of SPNpro’s PROTAC function in various cancer cells (Figure 10). When SPNpro accumulates in the tumor of living mice, CatB cleaves SPNpro, releasing the IDO-targeting PROTAC peptide (IPP) [80,81,82]. This activation triggers the targeted proteolysis of the immunosuppressive indoleamine 2,3-dioxygenase (IDO) in tumor cells. The released PROTAC binds to IDO and brings it to the E3 ubiquitin ligase VHL, leading to the persistent degradation of IDO via the ubiquitin-proteasome system. This targeted proteolysis of IDO by SPNpro’s PROTAC function is essential for promoting the activation of effector T cells and enhancing antitumor T-cell immunity. Thus, it enhances antitumor T-cell immune responses, leading to the inhibition of tumor growth and prevention of metastasis [78,82]. Zheng and his colleagues synthesized three molecules, SPNpro, SPN1, and SPN2 (Figure 10). They used PCB-PEG-IPCP, PCB-PEG-IPC, and PCB-PEG-PCP polymers, respectively, and allowed self-assessment of particles that gave rise to the nanoparticles. Differences among the polymers used lie in the presence of the IDO-targeting unit and the VHL-binding unit. SPN1 and SPN2 are control nano-particle compounds, while SPNpro is the compound carrying the IDO-targeting and the VHL binding units, as shown in Figure 10 [79]. 

Moreover, SPNpro can be activated by photoirradiation as it generates singlet oxygen (^1^O_2_) to eliminate tumor cells. When exposed to near-infrared (NIR) photoirradiation, SPNpro produces singlet oxygen as detected using a singlet oxygen sensor green (SOSG) fluorescence indicator. The generation of ^1^O_2_ was confirmed by the gradual increment in SOSG fluorescence intensity at 520 nm under NIR photoirradiation. This singlet oxygen generation is a key mechanism by which SPNpro exerts its phototherapeutic effects to eliminate tumor cells. Furthermore, a research group took advantage of SPN and conjugated with NGL919 through a singlet oxygen (^1^O_2_) cleavable linker and not a CatB one [80]. Upon NIR laser irradiation, the SPN core generates both heat and ^1^O_2_, which was detected by singlet oxygen sensor green (SOSG) as a fluorescence indicator. The generated ^1^O_2_ specifically cuts the linker to trigger the release and activation of NLG919, leading to proliferation and activation of effector T-cells but suppression of Treg cells.

## 9. Poly-PROTAC

**Figure 11:**
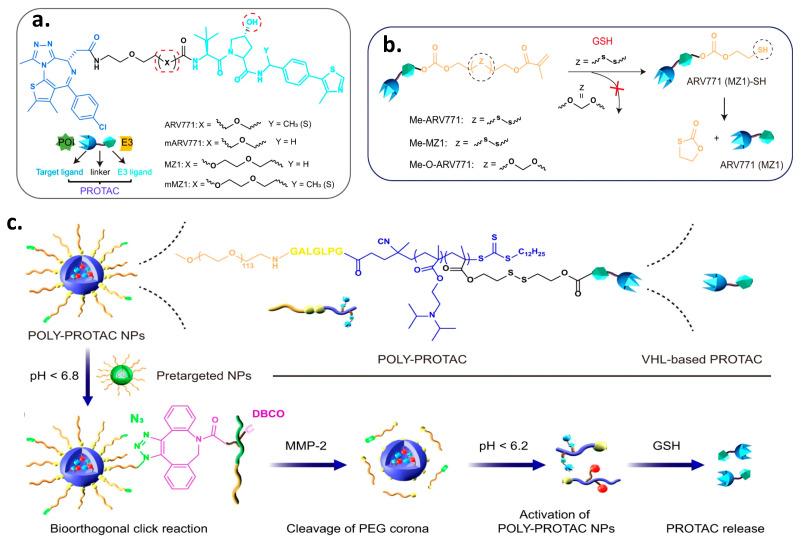
(**a**) Structure of the BRD4-targeted VHL PROTACs; (**b**) Schematic illustration of the GSH-triggered activation of ARV771 from Me-ARV771; (**c**) Azide-functionalized bioorthogonal POLY-PROTAC NPs prepared by grafting an MMP-2-liable PEG chain, an acid-activatable DPA moiety, and a reduction-sensitive disulfide spacer. When sequentially administered, a click reaction occurs between the POLY-PROTAC and DBCO-labelled pretargeted NPs, and the active PROTAC is only released at the tumor site. Figure adopted from *Nat Commun.* 2022 Jul 26;13(1):4318 [83] under creative common license.

Recently, Gao et al. reported the development of a series of polymer-based PROTACs (Poly-PROTACs) arranged into micellar nanoparticles to induce BRD4 degradation to provide precise cancer therapy [83]. They first prepared four VHL-based small molecular PROTACs with the ability to degrade BRD4 in MDA-MB-231 breast cancer cells in vitro. Then, the hydroxyl groups of the designed PROTACs, ARV771 and MZ1 were methacrylated with a disulfide linker to inhibit the interaction with the VHL protein and produce latent PROTACs activatable by reduction (Figure 11). Afterward, the target Poly-PROTACs were prepared via the technique of reversible addition-fragmentation chain transfer (RAFT) polymerization with the 2- (diisopropylamino)ethyl methacrylate (DPA) monomers. This allows the latent release of the active PROTAC when exposed to intracellular acidity (e.g., pH 5.5–6.5) and glutathione (GSH)-mediated reduction of the disulfide bond. Moreover, a matrix metalloproteinase-2 (MMP-2)-cleavable linker of seven peptides, GPLGLAG, has been inserted to achieve tumor-specific infiltration and enhance the uptake of the polymer. Finally, the nanoprecipitation method was used to prepare Poly-PROTAC spherical micellar NPs with an average diameter of ~55 nm. A series of POLY-PROTAC reduction-activatable compounds was synthesized that could self-assemble into micellar nanoparticles (NP). In addition, the authors developed acidity-activatable pretargeted NPs with the ability to dissociate in the acidic extracellular tumor environment. These pretargeted NPs were prepared by the self-assembly of dibenzocyclooctyne (DBCO)-modified mPEG-b-polyethylene propyl amine diblock copolymer. On the other hand, an azide group was added to the hydrophilic PEG head of the Poly-PROTAC NPs to allow the in situ biorthogonal click reaction with DBCO groups of the pretargeted NPs selectively at the tumor site [83].

The Poly-PROTAC NPs were i.v. administered 2 h after the DBCO-modified pretargeted NPs. This allowed the selective targeting of the tumor tissues, followed by sequential cleavage of the Poly-PROTACs by extracellular matrix metalloproteinase-2 and the intracellular acidic and reductive tumor microenvironment. Interestingly, these techniques showed several advantages over the conventional PROTAC approach, including (i) prolongation of the blood circulation time; (ii) higher tumor accumulation (3.9-fold) than their small molecule counterparts; (iii) the availability of several methods of activation (extracellular MMP-2, intracellular acidity, and reduction with glutathione) to release the active PROTACs. The study demonstrated that the administration of the small molecule PROTAC alone slightly suppressed the proliferation of MDA-MB-231 breast cancer cells in vitro. However, the combination of the bioorthogonal NPs dramatically inhibited 95% of tumor growth. Moreover, the co-administration of the pretargeted NPs with the Poly-PROTACs prolonged the survival of the tumor-bearing mice by 40% compared to those that received the Poly-PROTACs only. Collectively, these results highlight the great potential of this strategy for selective and prolonged delivery of the anti-cancer PROTACs to the tumor sites [83].

## 10. Challenges and Perspective

The utilization of PROTACs in drug development presents both promise and obstacles. While PROTACs offer a unique approach by targeting proteins for degradation rather than inhibition, several challenges impede their clinical translation. One significant hurdle is achieving specificity, as PROTACs must precisely recognize the target protein amidst a complex cellular environment to avoid off-target effects. Additionally, issues related to pharmacokinetics, such as stability and bioavailability, require careful optimization to ensure effective drug delivery. Furthermore, the potential for immunogenicity and toxicity remains a concern, necessitating thorough preclinical evaluation. Transforming the PROTAC into a prodrug can add to the complications of the situation, requiring additional synthetic steps and more careful optimization of the pharmacokinetic properties. Generally, the challenges facing the development of PROTAC prodrugs are related to three aspects of the drug discovery process: (i) medicinal chemistry, (ii) bioavailability and pharmacokinetics, (iii) in vivo activation [18,84,85,86]. First, the increased molecular weight, number of rotatable bonds, and number of functional groups entail long synthetic routes providing low overall yields. Therefore, the chemical reactions to install the caging group, mostly on a hotspot of the E3 ligase ligand, or to formulate the PROTAC into a micelle or a nanoparticle, should be optimized and incorporated into the synthesis workflow seamlessly. Second, the added molecular complexity and flexibility could negatively impact the ligand cell permeability and pharmacokinetics, as in the case of opto-PROTACs or deteriorate its solubility and absorption (e.g., SPNPRO). It is expected that molecules violating Lipinski’s rule of five to be less bioavailable and suffer from low aqueous solubility, poor cell permeability, and rapid clearance. However, the unique mechanism of action of PROTACs suggests a potential for adequate in vivo efficacy, justifying its investigation in clinical trials. PROTACs were found to rapidly and effectively degrade the POI, and small extra doses would be needed to deplete the target protein reservoir. Taking into consideration the slow gene expression required to restore the physiologically relevant concentration of several POIs, PROTACs could be active in vivo at low concentrations and long dosing intervals [84,87,88].

Finally, it is imperative to predict the off-target effects of not only the PROTAC but also its metabolites, including the “promoiety” upon its cleavage. Like with any prodrug, the rate of release of the active form of the PROTAC should be carefully studied and tailored to ensure the delivery of the appropriate therapeutic dose. For instance, the opto-PROTAC developed so far can only target skin and blood diseases due to the low tissue penetration of the activating UV radiation. On the other hand, prolonged exposure to these UV wavelengths may lead to DNA damage. Therefore, the development of novel caging groups that can be cleaved using near-IR radiation could open new avenues for the clinical applications of opto-PROTACs. Despite these challenges, the innovative mechanism of PROTACs holds immense potential for addressing previously undruggable targets and advancing precision medicine. The limitations in the scope of this review include the discussion of the toxicity that could be imparted by the “pro moiety” as well as the pharmacokinetic challenge resulting from increasing the bulkiness and, probably, the lipophilicity of the already large PROTAC molecule. These limitations, among others, will be the subject of our future mini-review.

## 11. Methods

To make our literature survey as comprehensive as possible, we performed two searches using the Reaxys database. First, we searched for the keyword “pro-PROTAC” in the publication titles or abstracts to retrieve 169 documents. Then, we searched for the more general term “PROTAC” and obtained 2040 original research titles, which were screened to retrieve any documents describing a PROTAC prodrug. The search was performed on 1 April 2025, and the review covers the prodrugs of PROTAC published till that date. Chemical structures were prepared using ACD Chemsketch Version 12 (Advanced Chemistry Development Inc., Toronto, ON, Canada).
